# Induction of *lacZ* Mutations in Muta™Mouse Primary Hepatocytes

**DOI:** 10.1002/em.20540

**Published:** 2010-05

**Authors:** Guosheng Chen, John Gingerich, Lynda Soper, George R Douglas, Paul A White

**Affiliations:** Environmental Health Sciences and Research Bureau, Research and Radiation Directorate, Health CanadaOttawa, Ontario, Canada

**Keywords:** primary hepatocytes, mutation, Muta™Mouse

## Abstract

We have developed an in vitro mutation assay using primary hepatocytes from the transgenic Muta™Mouse. Primary hepatocytes were isolated using a two-step perfusion method with purification by Percoll, cultured, and treated with benzo[*a*]pyrene (BaP), 2-amino-1-methyl-6-phenyl- imidazo[4,5-b]pyridine (PhIP), 3-nitrobenzoanthrone (3-NBA), and cigarette smoke condensate (CSC). The mean *lacZ* mutant frequency (MF) for the solvent control was approximately twofold greater than the spontaneous MF observed in liver tissue. A concentration-dependent increase in MF (up to 3.7-fold above control) was observed following exposure to BaP. Fourfold and twofold increases in mutant frequency were observed for 3-NBA and PhIP exposures, respectively, without the addition of any exogenous metabolic activation. A slight but statistically significant increase in *lacZ* MF was observed for CSC, but only at the lowest concentration. This is the first report demonstrating that mutations can be detected in cultured primary hepatocytes from Muta™Mouse. The preliminary results presented suggest that the Muta™Mouse primary hepatocyte mutagenicity assay can be used as a cost-effective tool for screening of environmental mutagens and therapeutic products. Environ. Mol. Mutagen. 51:330–337, 2010. © 2009 Wiley-Liss, Inc.

Transgenic rodent (TGR) mutation models such as Muta™Mouse and Big Blue® rat/mouse provide efficient methods for quantitative assessments of in vivo gene mutation. Such transgenic mutation assays involve scoring of mutations at transgenic *lacZ* or *lacI* sequences carried on a lambda phage shuttle vector that has been stably integrated into the rodent genome. The shuttle vectors containing the transgenic targets exist in every cell of the transgenic animal and are easily recovered from genomic DNA using a convenient in vitro packaging system [Gossen et al., 1989; Kohler et al., 1991; Douglas et al 1996]. A major advantage of the transgenic mutation system lies in its ability to provide reliable and reproducible assessments of in vivo mutagenicity in any organ or tissue [Heddle et al., 2000; Nohmi et al., 2000; Thybaud et al., 2003]. In their detailed review paper, Lambert et al. concluded that TGR mutation models showed excellent concordance (77%) with rodent carcinogenicity that meets or exceeds what has been observed for other genotoxicity assays commonly employed for regulatory decision-making (e.g., bone marrow micronuclei or unscheduled DNA synthesis in liver) [Thybaud et al., 2003; Lambert et al., 2005].

Although in vivo TGR mutagenicity assays offer the advantages of utility for regulatory screening, matching in vitro versions provide an opportunity for high-throughput analyses of test mutagens (e.g., new chemicals or drug candidates). A number of approaches have been employed to establish cell lines derived from TGRs. For example, a Big Blue® mouse embryonic fibroblast cell line was derived from primary embryo cells immortalized and transformed by X-ray irradiation and benzo[a]pyrene (BaP) exposure [Erexson et al., 1998]. BBR1 and BBM1 cells were derived from the primary skin fibroblasts of the Big Blue® rodents [Erexson et al., 1999]. Several epithelial and fibroblast cell lines have been derived from the rat mammary gland and oral cavity, and these cells were immortalized by exposure to the alkylating agent N-ethyl-N-nitrosourea [McDiarmid et al., 2001; Papp-Szabó et al., 2003]. Watanabe et al. [2001] established two mammary carcinoma cell lines derived from 2-amino-1-methyl-6-phenyl-imidazo[4,5-b]pyridine (PhIP)-induced Big Blue® rat mammary adenocarcinomas. Finally, a spontaneously immortalized epithelial cell line, known as FE1, was derived from Muta™Mouse lung tissue. The FE1 line has proved to be a useful tool for rapid and effective screening of environmental mutagens [White et al., 2003; Jacobsen et al., 2007, 2008a,b; Berndt-Weis et al., 2009].

The aforementioned cell lines, and indeed all cell lines derived from nonhepatic tissue, have a limited endogenous capacity to metabolize test mutagens. In general, transformed cell lines lose their capacity to metabolize or activate promutagens. Some researchers have even reported a lack of sensitivity for the widely used hepatic HepG2 cells, in comparison with primary human hepatocytes [Wilkening et al., 2003]. Consequently, an exogenous metabolic activation mixture (e.g., postmitochondrial supernatant from Aroclor-induced rat liver) is often required to permit Phase I metabolism and conversion of promutagens into reactive metabolites. For example, an exogenous S9 mixture from rat liver was required in a study that investigated the muta-genic activity of PhIP in the BBR/MFib fibroblast system [McDiarmid et al., 2002].

The liver is the primary organ for the metabolism of xenobiotic substances by Phase I and Phase II biotransformation enzymes. Cultured primary mammalian hepatocytes can retain the characteristics of liver cells and have been shown to contain a broad spectrum of xenobiotic metabolizing enzymes [Ulrich et al., 1995]. The metabolic capacity of cultured primary mammalian hepatocytes suggests that they should be ideal for the evaluation and screening of suspected environmental mutagens. Indeed, the utility of cultured primary hepatocytes has already been definitively demonstrated in general toxicology and

for early screening of drug candidates [Ulrich et al., 1995]. However, the established hepatic assays for genotoxicity screening (e.g., unscheduled DNA synthesis, DNA adducts/repair) do not require the property of cell proliferation [Casciano 2000].

In vitro gene mutation assays are generally carried out with continuously dividing cells, despite their distinct metabolic insufficiency. The lack of mitogenesis, and thus, the limited capacity for cell division of primary hepatocytes, has prevented their use for the scoring of gene mutations. However, recent advances in cell culture techniques can permit limited proliferation of primary hepatocytes, and primary hepatocyte cultures have been employed to assess induction of sister chromatid exchanges and micronuclei [Eckl and Raffelsberger, 1997; Müller-Tegethoff et al., 1997]. Several studies have shown that the addition of selected growth factors and hormones (e.g., insulin, epidermal growth factor [EGF] or hepatocyte growth factor [HGF]) can induce proliferation of primary hepatocytes in vitro [Matsumoto and Nakamura, 1991; Block et al., 1996; Müller-Tegethoff et al., 1997]. In this pilot study, we demonstrate that cultured primary hepatocytes derived from the Muta™Mouse can be employed to assess the mutagenic activity of selected test mutagens that require metabolic activation by cytochrome P450 isozymes.

## MATERIALS AND METHODS

### Materials and Reagents

All cell culture media and reagents were purchased from Sigma-Aldrich (Oakville, ON, Canada). 3-Nitrobenzoanthrone (3-NBA) was obtained from the Sigma Library of Rare Chemicals (Oakville, ON, Canada). BaP was obtained from Supelco Canada (Mississauga, ON, Canada) and PhIP was obtained from Toronto Research Chemicals (Downsview, ON, Canada). Preparation of the cigarette smoke condensate (CSC) was performed at Labstat International Inc. (Kitchener, ON, Canada). Combustion (i.e., smoking) of commercially available full flavor cigarettes was carried out on a 20-port rotary smoking machine (see Moir et al. [2008] for details). The smoking parameters and smoking machine specifications followed the International Organization for Standardization's standard ISO 3308 (i.e., Routine Analytical Cigarette-Smoking Machines Definitions and Standard Conditions) (see Moir et al. [2008]). Mainstream smoke was passed through a 92-mm glass fiber filter disc for particulate matter collection. To prepare tobacco smoke condensates, filter pads were placed in a flask containing dimethyl sulfoxide (DMSO) (ACS spectrophotometric grade, >99.9%) and shaken on a wrist-action shaker (Barnstead International, Melrose Park, IL) for 20 min. Each sample was standardized to a concentration of 30 mg total particulate material (TPM) per ml of DMSO.

### Transgenic Muta™Mouse

The transgenic Muta™Mouse (BALB/c × DBA2, mouse strain 40.6) was developed using a bacteriophage lambda shuttle vector containing the bacterial *lacZ* gene as a target for mutation detection [Gossen et al., 1989]. The transgenic mice were bred and maintained at Health Canada facilities under conditions approved by the Health Canada Animal Care Committee.

**Fig. 1:**
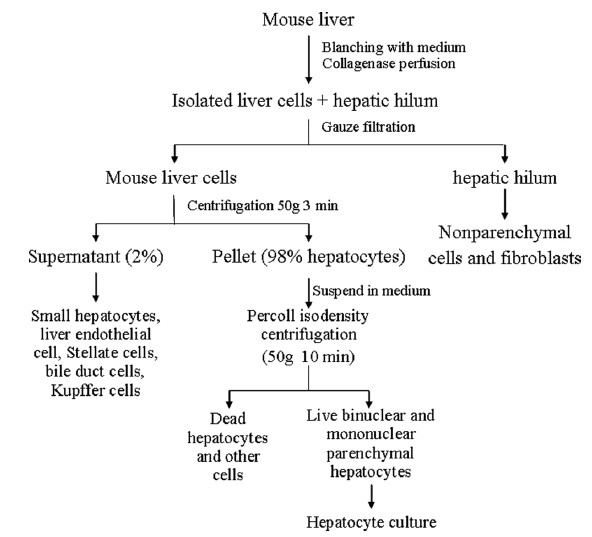
Scheme showing the procedure employed to isolate nearly pure hepatocytes via two-step perfusion and Percoll isodensity centrifugation (Adapted from the works of Seglen [1976], Kreamer et al. [1986], Block et al. [1996], Tateno et al. [2000], and Kruglov et al. [2002]).

### Isolation of Primary Hepatocyte Cell Culture and Chemical Treatment

Two 18–22-week-old male *lacZ* transgenic mice were used in this pilot study. Primary hepatocytes were isolated from Muta™Mouse by an adaptation of a two-step collagenase perfusion technique that involves enrichment prior to culturing using Percoll isodensity purification [Seglen, 1976; Kreamer et al., 1986; Tateno et al., 2000; Chen and Bunce, 2003]. A schematic of the procedure employed for isolation of parenchymal hepatocytes is provided in Figure 1. In brief, the mice were anesthetized by an i.p. injection of 100 mg/kg pentobarbital. The caudal vena cava was catheterized, the liver perfused with Hank's balanced salt solution (HBSS; pH 7.4, without Ca^2+^, Mg^2+^, 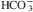, or phenol red) containing 1 mM ethylene glycol tetraacetic acid and 10 mM 4-(2-hydroxyethyl)-1-piperazineethanesulfonic acid (HEPES) for ∼2 min, followed by hepatocyte-qualified collagenase (0.3 mg/ml) in William's E medium (pH 7.4), supplemented with 10 mM HEPES and 0.1 mg/ml albumin, for ∼10 min. The digested liver was then excised, rinsed, and disaggregated in a 150-mm polystyrene Petri dish. The material was filtered through sterile gauze, and the filtrate was gently centrifuged for 3 min at 50*g*. The pellet was resuspended in 10 ml attachment medium (William's E medium supplemented with 10 mM HEPES, 2 mM l-gluta-mine, and 10% fetal bovine serum) combined with 10 ml of Percoll in HBSS and recentrifuged at 50*g* for 10 min. After the enrichment by Percoll isodensity purification, the cells were washed and gently centrifuged, and the pellets were resuspended in ∼20 ml of attachment media. The cells were counted using a hemocytometer. The viability of the cells was >90% as assessed by the trypan blue dye exclusion method.

The cells were placed (2.5 × 10^5^ cells/3.0 ml attachment media) in 60-mm polystyrene tissue culture dishes (Corning, Corning, NY) pre-coated with collagen. After 2 hr, the attachment medium was removed and 3.0 ml serum-free medium (William's E medium supplemented with 10 mM HEPES, 2 mM l-glutamine, 10 mM pyruvate, 0.35 mM proline, 20 units/l insulin, 100 units/ml penicillin G, 100 mg/ml streptomycin sulphate) containing 1 ng/ml murine EGF was added to each plate. The cells were then incubated at 37°C (95% relative humidity, 5% CO_2_). After 12 hr the cells were treated with various concentrations of test mutagens in serum-free medium containing 1 ng/ml EGF for 6 hr. After treatment, the cells were washed with phosphate-buffered saline (pH 7.6) and incubated in serum-free medium containing 1 ng/ml EGF for 48 hr before mutation scoring.

### Isolation of Genomic DNA

Genomic DNA was isolated as previously described [Vijg and Douglas, 1996; Douglas et al., 1999], with modifications for cultured cells [White et al., 2003]. Briefly, treated cells were digested overnight in lysis buffer at 37°C (10 mM Tris, pH 7.6, 150 mM NaCl, 10 mM ethylenediaminetetraacetic acid [EDTA], with 1% sodium dodecyl sulphate and 1 mg/ml fresh proteinase K), and lysates extracted with phenol/chloroform (1:1), followed by chloroform. Potassium acetate was added to a final concentration of 1.6 M and the DNA was precipitated in ethanol. DNA was spooled onto a sealed Pasteur pipette, washed with 70% ethanol, placed in 15–25 μl of Tris-EDTA buffer (10 mM Tris, pH 7.6, 0.1 mM EDTA), and stored at 4°C for further analysis.

### *lacZ* Mutant Frequency Analysis

Transgene mutant frequency (MF) was determined using the phenyl-β-d-galactopyranoside (P-gal)–positive selection assay [Vijg and Douglas, 1996; Lambert et al., 2005]. The method employs *galE*^−^ host bacteria to facilitate the isolation and enumeration of mutant copies of the *lacZ* transgene [Gossen et al., 1992]. λgt10*lacZ* DNA copies were rescued from genomic Muta™Mouse DNA (4 μl aliquots) using the Transpack™ lambda packaging system (Stratagene, La Jolla, CA). Packaged phage particles were mixed with host bacteria (*Escherichia coli* Δ*lacZ*^−^, *galE*^−^, *recA*^−^, pAA119 with *galT* and *galK*) [Gossen et al., 1992] and plated on minimal agar with 0.3% (w/v) P-gal. Concurrently, bacteria were plated on nonselective minimal agar to enumerate total plaque-forming units (pfu) or titer. All plates were incubated overnight at 37°C. MF was expressed as the ratio of mutant plaques to total pfu. The data presented are summaries across numerous experimental replicates. MF and pfu values are readily available from the corresponding author.

### Statistical Analysis

MF data were analyzed by Poisson regression using SAS version 9.1 (SAS Institute, Cary, NC), and the data were fit to the model log(*E*(*Y_i_*)) = log *t_i_* + β**x**_*i*_, where *E(Y_i_)* is the expected value for the *i*th observation, β is the vector of regressions coefficients, **x**_*i*_ is a vector of covariates for the *i*th observation, and *t_i_* is the offset variable used to account for differences in observation count period (i.e., pfu). The offset (i.e., natural log of pfu) was given a constant coefficient of 1.0 for each observation, and log-linear relationships between mutant count and test mutagen concentration were specified by a natural log link function. Type 1, or sequential analysis, was employed to examine the statistical significance of the chemical treatment, and custom contrasts were employed to evaluate the statistical significance of responses at selected concentrations. Custom contrasts were accomplished by specifying an *L* matrix, and computing statistics for pairwise comparisons based on the asymptotic chi-square distribution of the likelihood ratio.

## RESULTS

### Morphological Changes

**Fig. 2:**
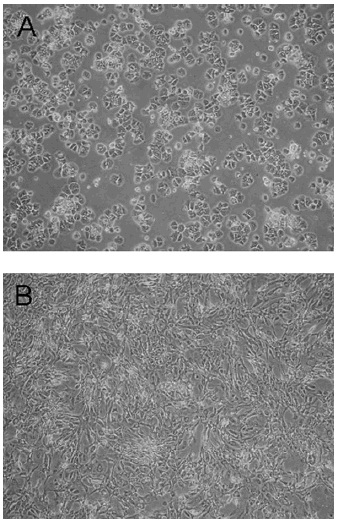
Phase-contrast photomicrographs of cultured primary hepatocytes. (**A**) Typical cubic hepatocytes shortly after isolation; (**B**) scattered, elongated hepatocytes after 48 hr (magnification, 40×).

Figure 2 illustrates the phenotypic changes of hepatocytes cultured in the presence of EGF. The freshly isolated mouse hepatocytes display typical cubic, nonproliferating morphology 2 hr after being plated (Fig. 2A). Both mononuclear and binuclear parenchymal hepatocytes were observed in the isolated cell populations. After 48 hr culture in serum-free medium supplemented with EGF, the hepatocytes display a more scattered proliferating morphology (Fig. 2B).

### Mutagenic Activity of Promutagens in Muta™Mouse Primary Hepatocytes

The overall MF for the vehicle control was 14.2 ± 5.6 × 10^−5^. This value is approximately twofold greater than that commonly observed in Muta™Mouse tissues (i.e., 5.9 × 10^−5^) [White et al., 2003]. In the initial pilot experiment (hepatocytes from two male mice), 1 ng/ml HGF was employed to stimulate hepatocyte growth, and the results revealed substantial inductions of *lacZ* mutants by 1.58 μM BaP (29.7 × 10^−5^) and 4 μM PhIP (30.8 × 10^−5^). These results confirmed the feasibility of the assay system. However, all subsequent experiments were conducted using EGF, a culture reagent that is far less expensive than HGF.

Muta™Mouse primary hepatocytes cultured in the presence of EGF were treated with several known environmental promutagens including BaP, PhIP, 3-NBA, and CSC. A summary of the MF values for these agents is presented in Table I. A concentration-dependent increase in MF (up to 3.7-fold above the concurrent control) was observed for primary hepatocytes exposed to BaP concentrations between 1.58 and 6.34 μM. A concentration-dependent increase in MF was also observed for PhIP, with the maximum response about 2.6-fold above control at the highest concentration tested (8 μM). At the low and middle concentrations (i.e., 1.45 and 3.63 μM), 3-NBA exposure induced a more than fourfold increase in MF; however, at the highest concentration (18.15 μM), cytotoxicity contributed to a low DNA recovery, and reliable MF scoring was not possible. For the CSC exposure, a statistically significant increase in MF was observed at the low concentration (80 μg TPM/ml); however, no significant increase was observed for the higher concentrations (i.e., 120, 160 μg/ml).

**Table I:** *lacZ* Mutant Frequency in Cultured Muta™Mouse Primary Hepatocytes

Chemicals	Concentration (μM)^a^	*n*^b^	Total mutants	Total plaques	Mean MF (×10^−5^)^c^	SD^d^	*P* value^e^
Solvent control	0	11	66	503,461	14.2	5.6	
BaP	1.58	5	78	286,935	27.5	11.3	0.0006
	3.17	5	71	217,852	35.4	17.9	<0.0001
	6.34	5	149	286,106	53.1	8.0	<0.0001
Poisson regression chi-square for test mutagen concentration effect = 61.7, *P* < 0.0001							
PhIP	2.0	5	38	168,483	22.1	6.8	0.015
	4.0	5	75	241,211	30.7	5.9	<0.0001
	8.0	5	39	130,380	36.8	17.5	0.0002
Poisson regression chi-square for test mutagen concentration effect = 28.2, *P* < 0.0001							
3-NBA	1.45	5	62	119,777	56.6	14.8	<0.0001
	3.63	3	26	32,139	79.4	19.5	<0.0001
Poisson regression chi-square for test mutagen concentration effect = 70.6, *P* < 0.0001							
CSC	80	5	46	198,966	23.1	6.6	0.005
	120	5	44	300,188	15.1	7.4	NS^f^
	160	4	21	110,831	18.8	4.5	NS
Poisson regression chi-square for test mutagen concentration effect = 8.8, *P* = 0.03							

a All concentrations in μM, except CSC, which is expressed as μg TPM/ml.

b *n*, the number of assays for mutation scoring.

c Mean *lacZ* mutant frequency per 10^5^ pfu.

d SD, standard deviation of the mean.

e Poisson regression with custom contrasts against the solvent control.

f NS, not significant.

## DISCUSSION

This study introduces a novel in vitro assay system for mutagenicity assessment that takes simultaneous advantage of the P-gal-positive selection system to score mutations at the *lacZ* transgene, and the metabolic capacity of primary hepatocytes. Although hepatocytes have a limited capacity for cell proliferation, EGF supplementation was employed to stimulate growth and division, and microscopic observations showed cell elongation and proliferation. Earlier works by Ichihara et al. [1982] and Nakamura and Ichihara [1985] have shown that mature hepatocytes, which are usually quiescent, will synthesize DNA and show density-dependent growth when cultured in the presence of insulin and EGF. Moreover, the work by Müller-Tegethoff et al. demonstrated the utility of cultured primary hepatocytes for the assay of micronuclei [Müller-Tegethoff et al., 1997]. This work extends the application of hepatocytes for genetic toxicity assessment.

Hepatocytes are the main functional liver cells, and they make up at least 60% of the cytoplasmic mass of the liver [Seglen, 1976]. In addition to hepatocytes, liver tissue contains endothelial cells, bile duct cells, Stellate cells, Kupffer cells, as well as supporting tissues. The standard two-step collagenase perfusion method employed in this study has been shown to yield 98% parenchymal hepatocytes, and the remaining 2% consisting of nonpar-enchymal cells (e.g., endothelial cells, bile duct cells, Stellate cells, Kupffer cells) can be separated by centrifu-gation (Fig. 1) [Seglen, 1976; Block et al., 1996; Tateno et al., 2000]. In our experiment, the isolated cells showed a homogeneous cubic morphology typical of nonproliferating hepatocytes (Fig. 2A); however, after 48 hr incubation with EGF the cultured cells displayed a scattered morphology (Fig. 2B), and this morphology is consistent with the observations of Block et al. [1996]. Moreover, Tateno et al. have shown that isolated hepatocytes, such as those shown in Figure 2, can be highly heterogeneous with respect to size and proliferation potential [Tateno et al., 2000]. Although it is possible that some fibroblasts coexist with the isolated hepatocytes and contribute to the measured MF values, it is important to note that liver fibroblasts are present mainly in the hepatic hilum, and the isolation of fibroblasts from the hilum requires the use of a different enzyme (i.e., pronase) [Kruglov et al., 2002]. Furthermore, the first centrifugation step employed in this study will effectively separate fibroblasts from hepatocytes. Thus, if fibroblasts are present in the isolated cell population, they would be expected to occur in trace amounts (see Fig. 1). Consequently, the isolated DNA employed for mutation scoring is mainly from hepatocytes, and not fibroblast contamination. Nevertheless, subsequent analyses should employ biochemical methods to investigate the composition of the isolated cell population (see discussion below).

BaP has been frequently used as a prototypical promu-tagenic carcinogen, and the observed increase in MF in the cultured Muta™Mouse hepatocytes employed in this study (i.e., 3.7-fold) is consistent with the results of in vivo studies. Although liver is not necessarily the target organ for BaP-induced neoplasia, up to a fivefold increase of *lacZ* gene MF (i.e., 22 × 10^−5^ vs. 4.1 × 10^−5^ in corn oil control) has been observed in the liver of Muta™Mouse orally exposed to BaP at 125 mg/kg/day for 5 days, followed by a 14-day manifestation time [Hakura et al., 1998]. And threefold induction of MF (i.e., 62.5 × 10^−5^ vs. 22.8 × 10^−5^ in the corn oil control) was seen in a subsequent study employing a 6-month manifestation period [Hakura et al., 1999]. Although differences in the exposure kinetics of in vitro and in vivo systems prohibit direct comparisons of MF values, the similarity in the trends highlights the utility of the in vitro system based on cultured primary hepatocytes.

PhIP, a heterocyclic aromatic amine identified in cooked foods, is a potent mutagen and animal carcinogen. Cytochrome P450 isozymes 1A1, 1A2, and 1B1 are believed to be involved in the metabolism and activation of PhIP via N-hydroxylation, followed by esterification to form *N*-acetoxy-PhIP that ultimately yields the highly reactive nitrenium ion [Boobis et al., 1994; Crofts et al., 1998]. PhIP is mutagenic in Salmonella and induces DNA damage, gene mutations, and cytogenetic abnormalities in cultured mammalian cells in the presence of an exogenous S9 metabolic activation system [IARC, 1993; Felton et al., 1994]. It has been documented that PhIP induced increases in MF in the liver of transgenic animals [Lynch et al., 1996; Masumura et al., 1999; Klein et al., 2001]. Our earlier in vitro work with the Muta™Mouse FE1 epithelial cell line showed PhIP-induced increases in *lacZ* MF only in the presence of exogenous S9 [White et al., 2003]. In this study, a concentration-dependent increase in MF was observed in cultured Muta™Mouse hepatocytes exposed to PhIP, and the observed increase (i.e., 2.6-fold) is consistent with the aforementioned results.

3-NBA is one of the most potent mutagens isolated from diesel emission particulates. Metabolism and activation of 3-NBA in mammalian systems is complex and believed to involve nitroreduction by NAD(P)H:quinone oxidoreductase and/or xanthine oxidase [Arlt et al., 2005; Chen et al., 2008]. TGR mutagenicity assessments revealed up to 4.8-fold induction in *cII* MF in Muta™Mouse liver after intraperitoneal treatment with 3-NBA (25 mg/kg body weight, administered once per week for 4 weeks) [Arlt et al., 2004]. Similarly, our earlier work showed a 4.2-fold induction in *lacZ* mutant frequency in Muta™Mouse liver following oral administration of 2 mg/kg/day for 28 days [Chen et al., 2008]. The increase in *lacZ* gene MF observed in this study (i.e., 5.6-fold) is consistent with these results.

Tobacco smoke is the most extreme example of a “systemic human mutagen” [DeMarini, 2004]. CSC has been shown to induce mutations at the *tk* locus in mouse lym-phoma cells [Clive et al., 1979] and *Hprt* mutations in CHO cells [Jongen et al., 1985] in the presence of exogenous metabolic activation. *Hprt* mutations have also been observed in a human lymphoblastoid cell line (MCL-5) that carries two recombinant plasmids expressing xenobiotic metabolizing enzymes [Krause et al., 1999]. There are more than 60 carcinogens in cigarette smoke, including several tobacco-specific nitrosamines such as 4-(methylnitrosamino)-1-(3-pyridyl)-1-butanone and *N*′-nitrosonornicotine, several polycyclic aromatic hydrocarbons (e.g., BaP), and aromatic amines such as 4-aminobiphenyl [Hecht, 2003]. Numerous studies have established that these carcinogens require metabolic activation by several cytochrome P450 isozymes [Hecht, 2008]. The results obtained here (Table I) demonstrate that 80 lg TPM/ml induced a significant increase in *lacZ* MF in cultured Muta™Mouse hepatocytes. The lack of mutagenicity observed at higher concentrations (i.e., 120, 160 μg/ml) was likely the result of cytotoxicity, which was evidenced by reduced cell survival and low yield of extractable DNA.

The results presented here confirm the utility of a *lacZ* gene mutation assay in cultured primary hepatocytes derived from the Muta™Mouse for in vitro screening of suspected environmental mutagens. This in vitro mammalian cell assay system has several noteworthy advantages: (1) significant reduction in the number of animals required for mutagen screening relative to in vivo studies; (2) the metabolic competence of primary hepatocytes and concomitant ability to metabolize and activate several types of promutagens; (3) nearly pure populations of fresh hepatocytes are relatively easy to obtain and culture; (4) the Muta™Mouse system for scoring *lacZ* mutations is well established and validated. In addition, the use of primary hepatocytes readily permits comparisons of the metabolism of chemicals across species, thus increasing the confidence of extrapolations from animals to humans [NRC, 2007].

Nevertheless, it should be noted that this work constitutes a pilot study, and follow-up work will be required to refine, validate, and optimize an assay based on cultured primary hepatocytes. There are numerous avenues for follow-up research. First, subsequent analyses should rigorously investigate the composition of the isolated cell population. To this end, biochemical tools, such as those described by Modriansky et al. [2000], could be employed to provide an enzymatic and proteomic profile of the isolated cells (e.g., total cytochrome P450 content, activity of selected P450 isozymes). As fibroblasts can proliferate in the absence of EGF, experiments conducted both in the presence and in the absence of EGF could permit an assessment of fibroblast contamination. Second, subsequent analyses should refine and optimize the assay protocol. For example, the magnitude and reproducibility of the response to selected mutagens could be assessed for cell populations derived from numerous animals, including very young animals (e.g., 14 days), as well as animals exposed to chemical inducers of liver enzymes (e.g., Aroclor). The former would be expected to maximize the proliferation potential of the isolated cells, and the latter would be expected to increase the metabolic capacity of the isolated cells. In addition, a larger culture surface could be employed to permit an increase in the number of exposed cells. Finally, subsequent analyses could employ established cytotoxicity-assessment tools (e.g., clonal survival) to reliably quantify effects that prevent or retard cell growth and proliferation.

In summary, we have developed and introduced an in vitro mutation bioassay based on cultured primary hepatocytes from the transgenic Muta™Mouse, and preliminary results indicate that the assay can be employed as a cost-effective complement to in vivo analyses for screening of environmental mutagens. The assay system can quantify mutations at the transgenic *lacZ* locus (this work), as well as the smaller *cII* locus. The latter can be more readily subjected to sequence analysis. Moreover, these endpoints can readily be combined with other genotoxicity end-points, including DNA strand breaks (i.e., comet), micronucleus formation, and unscheduled DNA synthesis.

## Abbreviations

BaP: benzo[*a*]pyrene
CSC: cigarette smoke condensate
DMSO: dimethyl sulfoxide
EDTA: ethylenediaminetetraacetic acid
EGF: epidermal growth factor
HBSS: Hank's balanced salt solution
HEPES: 4-(2-hydroxyethyl)-1-piperazineethanesulfonic acid
HGF: hepatocyte growth factor
MF: mutant frequency
3-NBA: 3-nitrobenzoanthrone
pfu: plaque-forming units
P-gal: phenyl-β-d-galactopyranoside
PhIP: 2-amino-1-methyl-6-phenyl-imidazo[4,5-b]pyridine
TGR: transgenic rodent
TPM: total particulate material
